# Application of Mobile Mapping System to a Cable-Stayed Bridge in Thailand

**DOI:** 10.3390/s22249625

**Published:** 2022-12-08

**Authors:** Ekarin Lueangvilai, Taweep Chaisomphob

**Affiliations:** School of Civil Engineering and Technology, Sirindhorn International Institute of Technology, Pathum Thani 12120, Thailand

**Keywords:** structural health monitoring, mobile mapping, real-time kinematic survey, rutting, IRI, elevation

## Abstract

Infrastructures must be inspected regularly to ensure serviceability and public safety. In the case of the Thailand expressway, 200 km of an elevated structure must be inspected once a year. Thailand expressway is an elevated reinforced concrete structure. Visual inspection for defects and structural movements such as excessive deflections, transverse movements, or settlements is a cumbersome process. Therefore, a mobile mapping 3D laser scanning (MLS) which is a high-resolution 3D laser scanner (Trimble MX-8) equipped on a vehicle, was introduced. Scanning was performed on live traffic on the expressway. From MLS, both the structure geometry and pavement point cloud data were obtained. A good agreement between elevations of the Rama XI bridge in Bangkok measured by point cloud data using MLS and by a real-time kinematic survey was obtained. The effect of mesh size on the output by MLS was investigated. It was found that a mesh size of 10 cm reduced the computational effort by 75% when compared to a mesh size of 5 cm. However, the International Roughness Index was reduced by 5%. International Roughness Index (IRI) estimated by MLS was close to the IRI values measured by the profilometer. However, a significant overestimation in the case of rutting depth was observed.

## 1. Introduction

Bridges are one of the most important constituents and a vital part of a country’s infrastructure. The health of an aging bridge needs to be assessed with fast yet effective methods for the sake of updated load limits that a bridge can sustain without accumulating extensive damage. Further, the knowledge of a bridge condition can help in devising effective maintenance programs to maintain or improve the declining condition. Cracks formed in a bridge can have a substantially detrimental effect: ranging from the reduction in load capacity to the change in dynamic characteristics of the bridge. The design of a bridge is carried out for a particular traffic limit. However, the rapid expansion of modern cities around the globe has seen a dramatic increase in the number of vehicles. In 2008, a report by the American Association of State Highway and Transportation Officials (AASHTO) stated that truck miles over existing bridges nearly doubled in two decades [1].

The visual inspection of bridges for the sake of their health assessment has been the most primitive method. The Federal Highway Administration (FHWA) recommends that the cycle of visual inspection for existing bridges should be repeated after every two years [2]. However, health assessment by visual inspection can only be considered an initial step toward detailed monitoring. This is because defects that are invisible to the naked eye cannot be detected by visual inspection. In addition, considerable variability within the results of visual inspection has been found in the past. For instance, Moore et al. [3] compared the visual inspection ratings of the same bridges estimated by different inspectors. Surprisingly, an average of four to five different ratings were assigned to the same bridge. Phares [4] stated a key disadvantage of visual inspection: the method could only work when damage endangers the structure’s life. Rehman et al. [5] suggested combining the visual inspection method with other advanced methods for a bridge health assessment.

The measurement of the global response of a bridge has the potential to reveal damage and defects. The idea is to obtain the global response in terms of velocity or acceleration by attaching several sensors to the bridge. From the measured vibrations, it is possible to extract modal characteristics of the bridge, such as natural frequency, damping ratio, or mode shapes. These modal characteristics correspond to a deteriorated bridge and can be compared to the modal characteristics of the same bridge in healthy conditions (usually obtained from FEM) to assess the extent of deterioration. Structural health monitoring (SHM) in this realm can be performed either by forced or ambient vibrations. The forced vibrations are disruptive in nature but accompany lower magnitudes of noise [6,7]. On the contrary, SHM via ambient vibrations is less disruptive for the traffic. However, the installation and management of various sensors and their wires is a clumsy task [8]. Ambient vibrations are polluted by significant noise levels and need a thorough understanding of signal processing techniques to denoise them. Further, an assumption of the stationarity of the captured signal is often made during its processing. Studies have shown that dynamic nature and structural faults often invalidate this assumption [9,10]. Bridge health assessment using non-destructive methods has emerged as a viable solution to primitive visual inspection in the last three decades. Breña et al. [11] conducted live load tests on a simply supported bridge. The resulting strains were measured. A finite element model (FEM) was created for the bridge, and strains within the bridge were measured using similar loading details. Chajes et al. [12] concluded that non-destructive testing aims to locate the damage at the material level. Subsequently, this type of health assessment is suitable in the case of concentrated damage.

Until now, a 3D terrestrial laser scanner (TLS) has been introduced for deformation monitoring of different applications in the field of survey engineering. Lovas et al. [13] studied the possibilities of a laser scanner for deformation monitoring of the Pentele bridge. The deformation monitoring of the Felsenau viaduct by using TLS was performed by Zogg et al. [14]. The results from TLS were then compared with the precise leveling method. Truong-Hong et al. [15] presented a framework for bridge inspection using TLS data. Although TLS can provide point cloud data with millimeters precision, these are often needed to be installed within the proximity of bridge lanes. As a result, the traffic flow over the bridge can be affected [16]. In recent years, mobile mapping systems (MLMs) have emerged as an efficient and time-saving technique for SHM purposes. Efficient field surveys of large areas can be conducted without disrupting the traffic flow. MLMs utilize trajectory information obtained from the Global Navigation Satellite System (GNSS) to establish point clouds in a common frame [16]. Erdélyi et al. [17] performed deformation monitoring of Liberty Bridge using TLS and ground-based radar interferometry. Both methods were found to be accurate and highly effective to analyze the deformation of engineering structures. Harmening and Neuner [18] developed a spatio-temporal deformation model by combining B-spline surfaces with stochastic modeling. The proposed modeling strategy was found effective in filtering datasets and predicting unmeasured values. In addition, different types of scanning techniques have been used in the past for remote sensing bridge deformations [19,20,21].

Infrastructure must be inspected regularly to ensure serviceability and public safety. In the case of the Thailand expressway, 200 km of the elevated structure must be inspected once a year. The present study investigates the performance of an MLS system for monitoring the Thailand expressway. Mobile mapping laser scanning (MLS) also provides pavement point cloud data. Pavement is normally checked for International Roughness Index (IRI) and rut depth which is calculated from pavement geometry. Every road agency in Thailand relies on a laser profilometer as a default machine. MLS point cloud data could be used for IRI calculation as well. So, expressway point cloud data were extracted for only pavement. Each pavement segment was executed for IRI and rut depth using the “Pavement inspection program” which is the program developed by Tokyo University and Shotoku engineering of Japan. A correlation between these two methods of IRI measurement was observed. The use of MLS is new in Thailand. The performances of MLS need to be examined. The procedure of measurement, data handling, data accuracy, and data exportation will be studied to achieve the monitoring by MLS.

The present study aims at the following objectives: (1) To use MLS point cloud data for structure deformation measurement. GPS position and elevation accuracy will be examined to obtain the accuracy of measurement. In Thailand, the existing historical bridges were constructed using truss and trestle-type structures such as Memorial Bridge (Bangkok), River Kwai Bridge, and Uttamanusorn Bridge. During the last decade, cable-stayed bridges have been extensively constructed across the main Chaophraya River at different locations. Among cable-stayed bridges, Kanchanaphisek Bridge is the longest bridge with a span of 500 mm followed by the Rama IX Bridge which is 450 m long. In Thailand, usually traditional SHM is performed using accelerometers, linear variable transducers, and data loggers. In the case of long-span bridges, the use of long cables is not convenient and accurate to connect measuring devices with the data loggers. Therefore, in this study, finally, a cable-stayed bridge movement will be measured for the health monitoring proposed, and (2) the roughness of pavement will be calculated using MLS point cloud data. Then, the calculated roughness will be compared to the conventional method using a profilometer. Finally, the measurement of the International Roughness Index (IRI) by using MLS will be compared to the one obtained using conventional methods.

## 2. Mobile Mapping System

**Figure 1:**
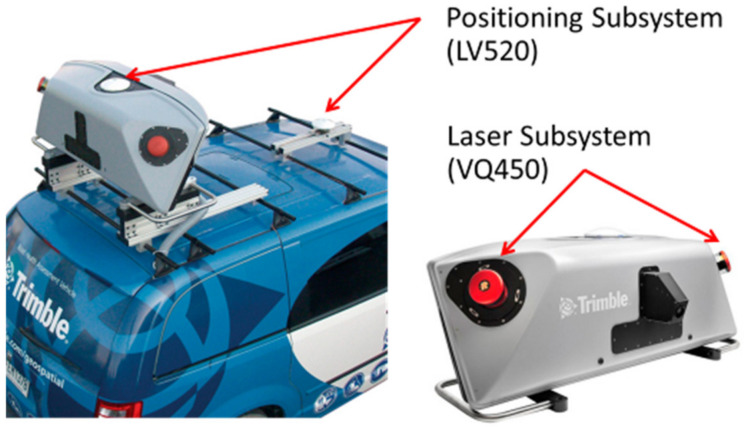
Mobile mapping 3D laser scanner (MLS).

The Metropolitan Expressway Company Limited: MEX, which is responsible for expressways in Tokyo, has introduced “InfraDoctor”. The InfraDoctor is a new web-based application for the Japanese expressway asset management system. It is aimed to extend the use of InfraDoctor in Thailand and Southeast Asia. The InfraDoctor is a web-based application for expressway asset management. The content of the website is 3D point cloud data of expressway and related documents such as drawings, inspection reports, and so on. The final development of the website is an online asset database that contains all information related to structure history on a geographic information system (GIS) platform. The main part of the InfraDoctor is 3D point cloud data of structures. The point cloud data are created from Mobile Mapping 3D Laser Scanner (MLS). The MLS is a vehicle equipped with 360° laser scanners, cameras, GPS antennas, and data loggers, as shown in Figure 1. The point cloud management program is “infiPoint™”. Each point in the point cloud contains values of coordinates in the UTM spatial reference system and color code in the RGB system.

**Figure 2:**
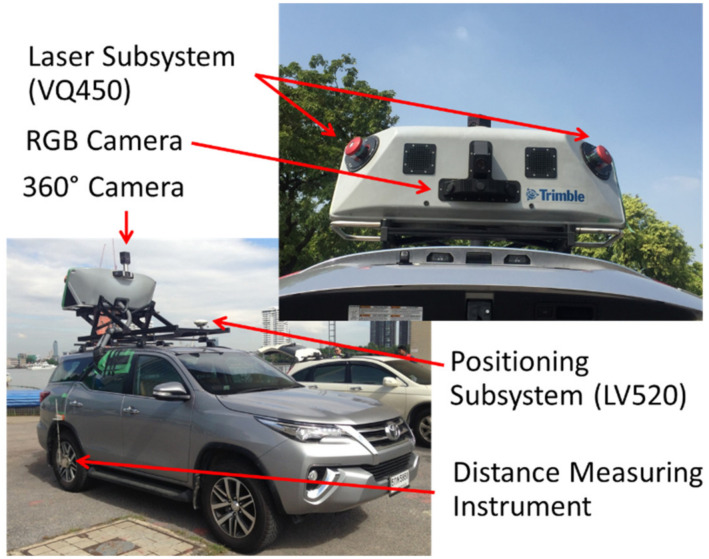
Details of MLS in current research work.

The MLS used in the current research work was Trimble MX-8, as shown in Figure 2. The MLS comprised GNNS, POS/LV520 model by APPLANIX. The laser scanners were VQ450 models manufactured by RIEGLE. A CCD camera was used, namely Grasshopper by Point Grey. A 360° camera was used, namely Ladybug 3 by Point Grey as well. The Trimble is a complete field-to-finish mobile mapping solution that combines leading-edge hardware with intuitive field software and a powerful, integrated office software workflow. Everything was equipped on a Toyota Fortuner model. The accuracy of the VQ450 laser scanner was 8 mm with a precision of 5 mm, as per the specifications. The measurement range was approximately 800 m. POS/LV520, which is a GNNS antenna, had an accuracy of 20 mm in X and Y directions, whereas this accuracy was 50 mm in the Z direction. The specifications of all aforementioned components were provided by the supplier.

**Figure 3:**
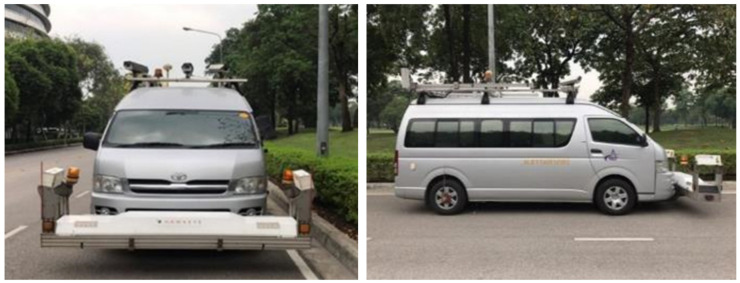
Profilometer attached in front of the MLS vehicle.

In addition to the measurement by MLS, the pavement of the expressway was also inspected. A profilometer was attached in front of the vehicle (see Figure 3) to facilitate the inspection of the pavement. The International Roughness Index (IRI) was measured by the laser profilometer. The accuracy of the estimated IRI by point cloud was assessed by comparing it to the IRI estimated by laser profilometer.

## 3. Research Methodology

**Figure 4:**
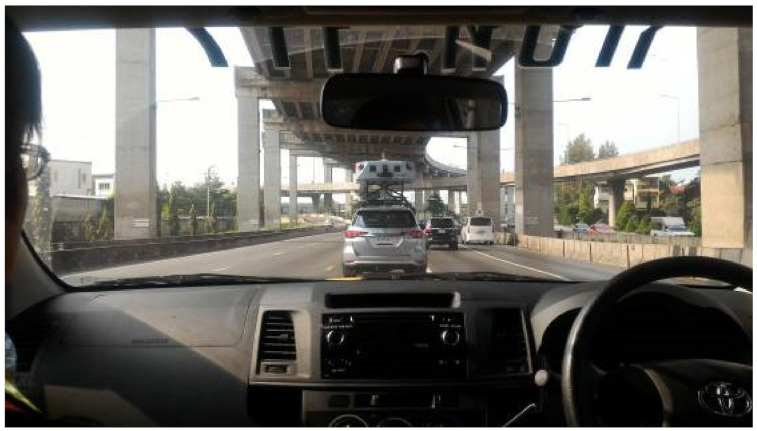
MLS scanning in operation.

Thailand expressways scanning was performed in December 2016. The scanning was performed at the RamaIX bridge in Bangkok. The base station was set at Chulalongkorn University, Bangkok and it was able to cover an area within a radius of 20 km. Within this radius, it was possible to cover the maximum municipal area of Bangkok city. On the expressway, the MLS measurement range was set at 100 m with a measurement rate of 0.5 MHz per laser head. Data were acquired at 1 million points per second. A shadow car was provided at a 5 m to 6 m interval to prevent another vehicle from cutting the scanning line. Figure 4 shows the MLS scanning in progress (taken from the shadow car).

**Figure 5:**
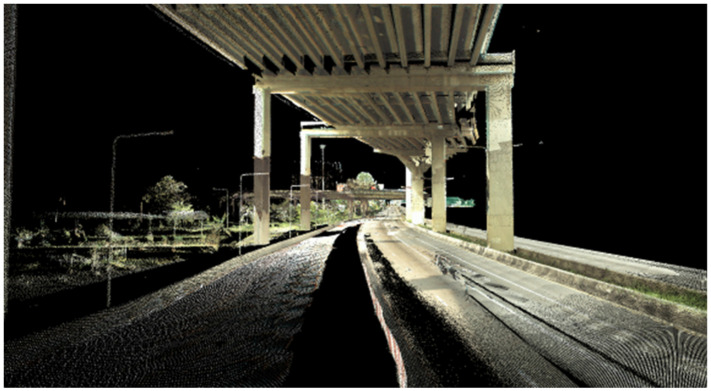
Pavement and expressway structure.

The speed of MLS can be up to 80 km/h. However, the speed was kept at 40 km/h due to safety concerns. The scanning was performed for two to three hours continuously. Then, a break was taken to allow the MLS to cool down. Point cloud data have been prepared by the machine operator (AERO Ashi Corporation, Koto City, Tokyo, Japan). Global coordinates were rectified with known coordinates. There were approximately 1900 points/square meter. The unwanted points, such as noise and irrelevant objects, such as vehicles or humans, were removed. The result was a point cloud of pavement and expressway structure without any vehicle, as shown in Figure 5. Two kilometers of road scanning produces up to 33.5 gigabytes (GB) of data. So, enough storage should be prepared in advance. The georeferencing was completed by RTK.

**Figure 6:**
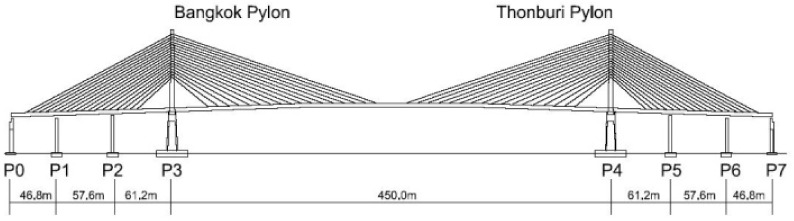
Profile of the bridge used in the present study.

**Figure 7:**
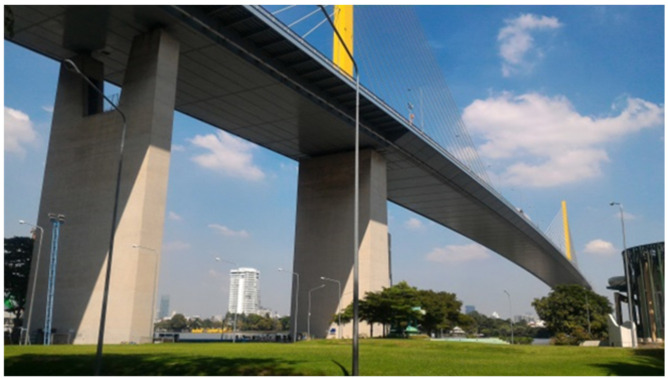
A view of Rama XI bridge.

In the present study, the measurements were performed on a 30-year-old single-span cable-stayed bridge. The bridge is named Rama XI bridge, accommodating six lanes of traffic. The total length of the bridge is 782 m, with a clear span of 450 m and the side spans consist of three sections with a length of 165.6 m each. The profile of the bridge is shown in Figure 6. The bridge decks comprised a three-cell steel box girder, 33 m wide and 4 m deep at the center. The 87-m-tall pylons pass through the bridge deck on the top of concrete piers. The steel pylons are closed rectangular boxes with additional internal framing to carry the cable anchorages. The piers that support the pylons have hollow concrete sections. The actual bridge is shown in Figure 7.

The bridge geometries, such as deck profile and pylon top movement, were measured according to the bridge inspection manual. The manual stated that the movement should not be larger than span/1000. Thus, the deflection of the 450 m span should not be greater than 450 mm. The lateral movement on the top of the pylon should not be larger than 87 mm. Normally, movement is measured by total station. Only five points on the bridge deck were measured in 2001, and thirty-four points on the bridge were measured in 2012, while pylon top movement has never been checked. Those measurements were performed using total station theodolite. Although total station theodolite is proven to be very successful for measurements, there is still a need to use relatively modern measurement techniques to examine bridge geometry change. Once point cloud data of the bridge was acquired, overall bridge geometry was acquired as well. However, elevation measured by point cloud needs to be validated first.

**Figure 8:**
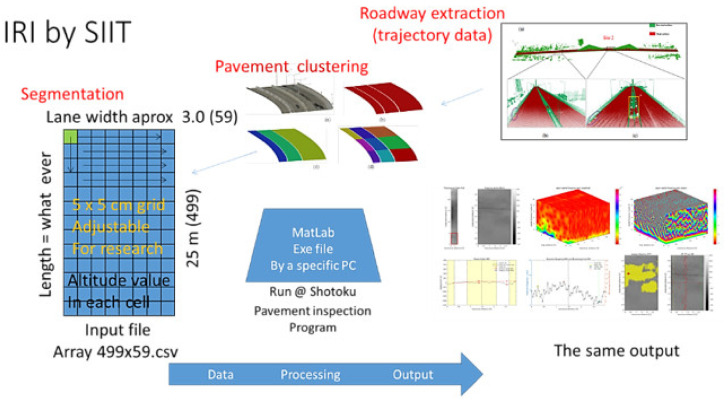
Working process for Expressway IRI calculation.

**Figure 9:**
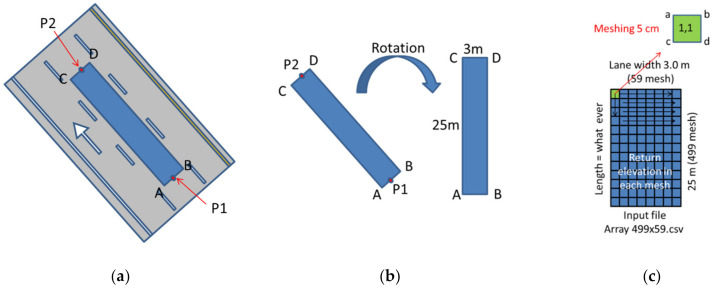
(**a**) Cropped area of the road segment, (**b**) rotation performed for the ease of meshing, and (**c**) cropped area meshing.

In order to verify IRI calculation by the two methods, i.e., by point cloud and laser profilometer, the data on the same piece of pavement must be extracted. IRI data from laser profilometer normally come with GPS coordinates. The GPS (originally known as NAVSTAR GPS) positions were used to extract point clouds. The width of the extracted pavement can be adjusted up to 3.5 m, according to the Thailand expressway lane width. The length of extracted pavement was 25 m. The working steps involved in the estimation of IRI are shown in Figure 8. These steps are summarized as follows: (1) all point clouds are extracted along the desired length of the road using either “infiPoints” or “Cloud Compare” software. In this research case, point cloud data of 1.6 km for six-lane expressways were extracted. This data comprised about 96 million points, and the corresponding file size was about 12 GB. A voxel-based region growing method was used for road surface extraction (2) the GPS data from the profilometer, points P1 and P2, was used to create the cropped area (Area A–B–C–D), as shown in Figure 9a. The width of the cropped area (A–B) was set at 3.0 m to 3.5 m depending on lane width. The length of the cropped area was dependent on the distance between points P1 and P2. So, the cropped area was 3.0 m × 25.0 m, according to profilometer GPS data. Then, point clouds were selected only in the “Cropped area”. The point density was about 1900 points/square meter, (3) the data were rotated using a built-in option to a perpendicular direction for ease of meshing, as shown in Figure 9b, and (4) meshing was performed on the cropped area. The smallest mesh size was kept at 5 cm. The point clouds in the mesh return the average elevation of a single mesh point. There were about 1900 points in a square meter. For a mesh size of 5 cm × 5 cm, there were roughly 4.82 points on average. These points were considered enough to obtain the average elevation. After this, the algorithm was shifted to the next mesh element.

## 4. Experimental Results

### 4.1. Validation of Bridge Profile Measurement

**Figure 10:**
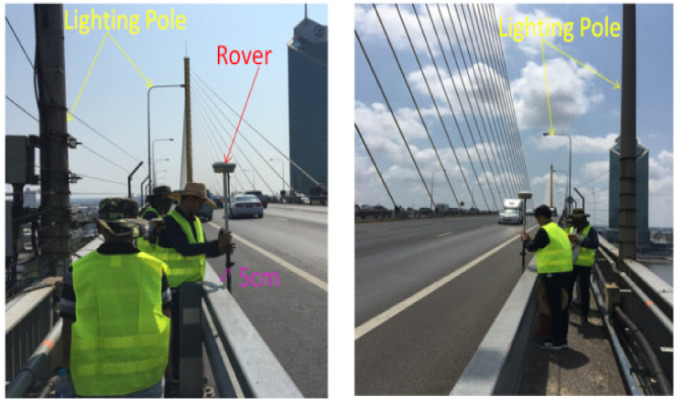
A real-time kinematic survey in progress.

A real-time kinematic survey, RTK (South S82T model), was used to validate the point cloud data. From provided specifications, the horizontal accuracy was ±8 mm, whereas the vertical accuracy was ±15 mm. Elevations were measured by the RTK for 66 points, as shown in Figure 10 (left: 20 points, right: 20 points, center: 24 points, and 1 point each on top of each pylon).

**Figure 11:**
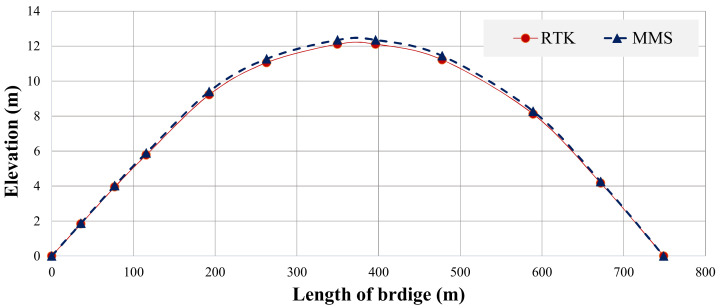
Comparison of bridge profile measured by RTK and MLS.

The bridge profile between MLS and RTK surveys are plotted together, as shown in Figure 11. The bridge profile values are also summarized in Table 1. It can be seen that a close agreement between the bridge profiles measured using RTK and MLS exists, validating the accuracy of MLS.

### 4.2. Bridge Deck Twist Angle

**Figure 12:**
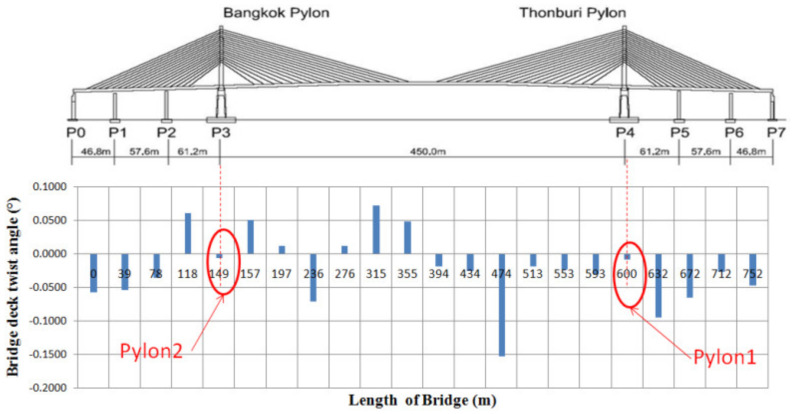
Measured twist angles of bridge by MLS.

Based on the MLS data, the twist of the bridge deck was examined. By comparing elevations between each side of the deck, twist angles were obtained. Note that elevations were measured on asphalt pavement. By deviation of the pavement thickness (20–30 mm) and elevation measurement accuracy (±15 mm), the measurement of twist angle with ±0.05° range was possible, assuming that the deck is perfectly horizontal. The results show that most of the measured twist angles were within the range (±0.05°), as shown in Figure 12. Angles at the pylons were low as expected. Twist occurred in both clockwise and counterclockwise directions. It can be concluded that no sign of a permanent twist was observed for the deck.

### 4.3. Pylon Deflections

**Figure 13:**
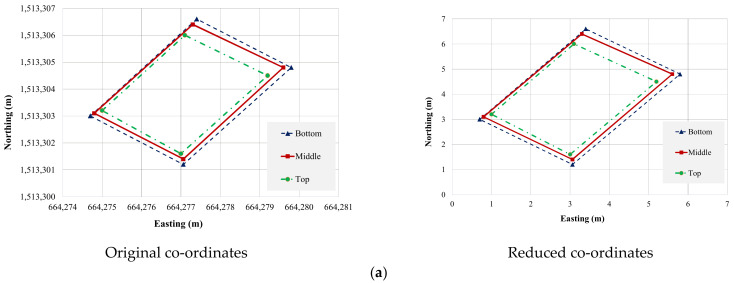

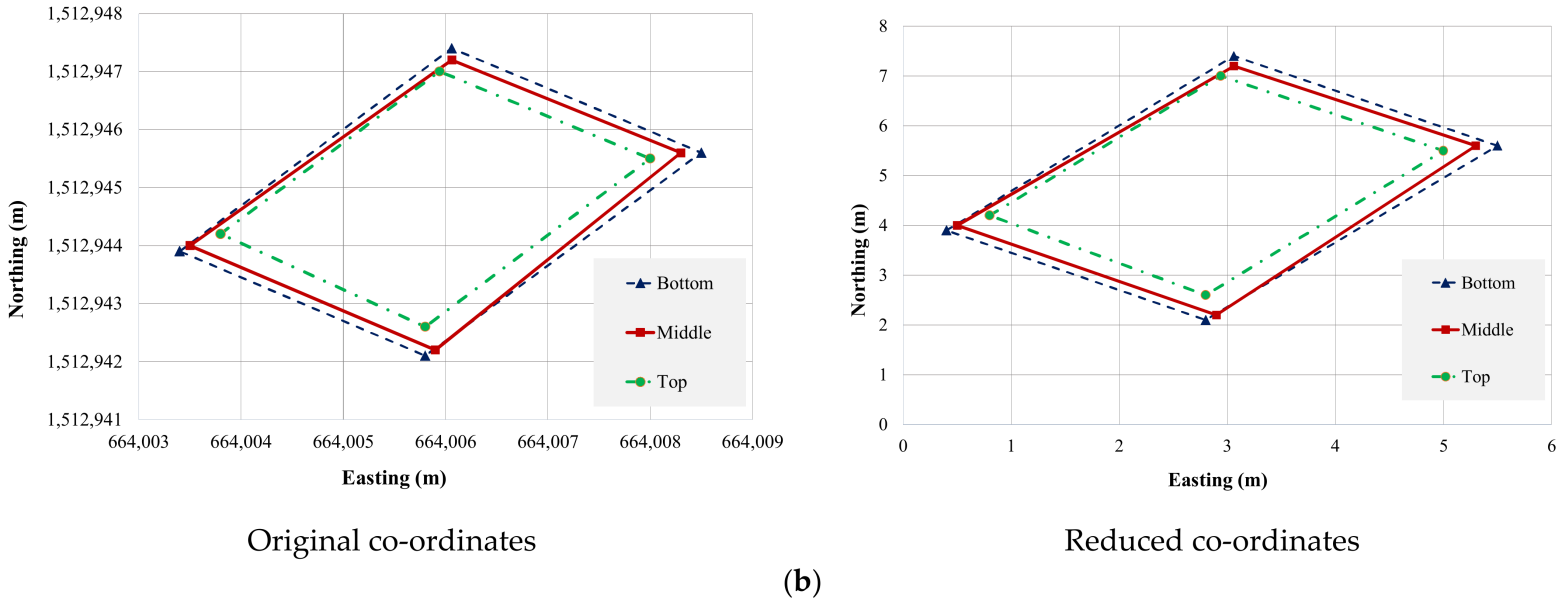
(**a**) East pylon section comparisons, and (**b**) west pylon section comparison.

**Figure 14:**
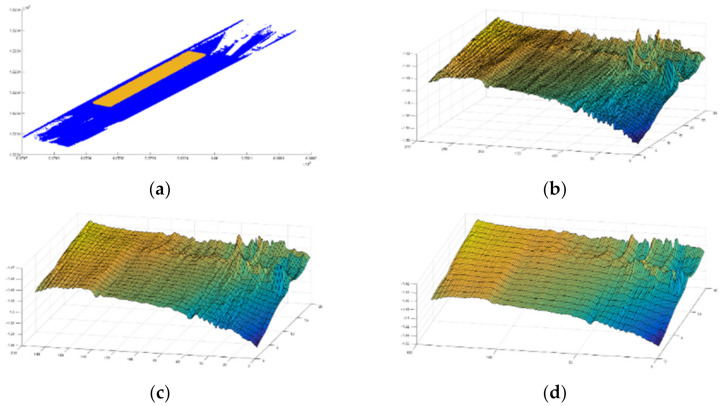
(**a**) Cropped area, (**b**) 10 cm × 10 cm mesh size, (**c**) 15 cm × 15 cm mesh size, and (**d**) 20 cm × 20 cm mesh size.

Pylons are not prismatic members. The size of the pylon was 3.0 m × 4.5 m at the bottom and 2.5 m × 3.5 m at the top. The point cloud data of the pylon was divided into three sections, bottom, middle, and top, using the InfiPoints. To check the top movements, the coordinates of each section were plotted together, and the position of their centroids was compared, as shown in Figure 13 and Figure 14. The movement was calculated with the assumption that the pylon was initially vertical. The results show that the difference between the actual positions and the ideal positions was 104 mm for the west pylon in the transverse direction and 138 mm for the east pylon in the longitudinal direction. These values were higher than the maximum value stated in the inspection manual. This could be due to many reasons such as noise and other factors. However, periodic monitoring was recommended since the position of the pylon after construction was not available.

### 4.4. Effect of Mesh Size on Measured International Roughness Index

As presented earlier, a minimum mesh size of 5 cm × 5 cm was used. However, to reduce the computational effort without affecting the measured IRI accuracy, mesh sizes of 10 cm × 10 cm, 15 cm × 15 cm, and 20 cm × 20 cm were studied, as shown in Figure 14. To assess the accuracy of different mesh sizes, the finest mesh size, i.e., 5 cm × 5 cm, was used as a reference.

**Figure 15:**
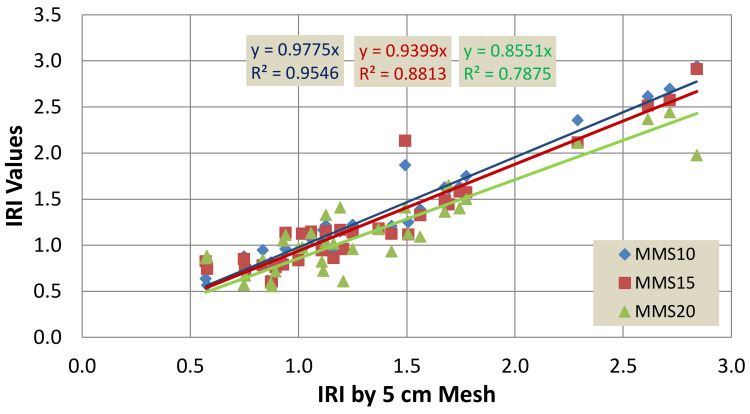
Comparison of the measured IRI using different mesh sizes with the measured IRI using a mesh size of 5 cm × 5 cm.

Figure 15 presents a comparison of IRI values computed by different mesh sizes with the mesh size of 5 cm × 5 cm. It can be seen that the best correlation with the mesh size of 5 cm × 5 cm was produced by the mesh size of 10 cm × 10 cm, as explained by the coefficient of determination of 0.99. The use of a large mesh size reduced the accuracy of the measured IRI. However, the reduction in the accuracy of measured IRI was not significant. In general, a linear trend was observed for all the mesh sizes, i.e., 5 cm × 5 cm, 10 cm × 10 cm and 20 cm × 20 cm. A precise reason is not yet clear. Further studies are required to investigate the proper reasons for the linear behavior.

**Figure 16:**
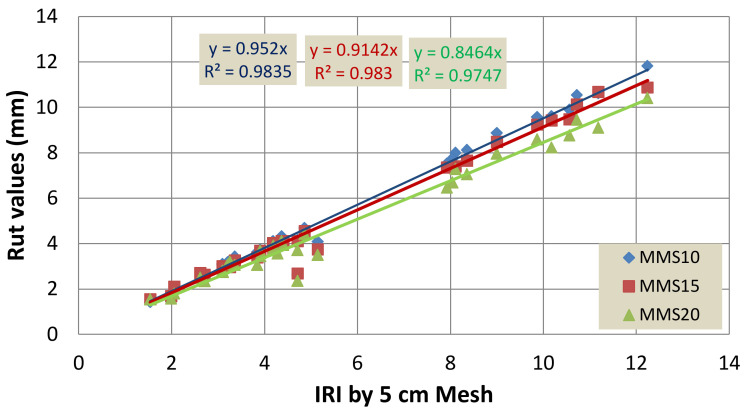
Comparison of the measured rutting depth using different mesh sizes with the measured rutting depth using a mesh size of 5 cm × 5 cm.

The rutting depth was also calculated using the point cloud data for various mesh sizes, with the mesh size of 5 cm × 5 cm as the reference. The resulting relation is plotted in Figure 16. The use of mesh sizes of 10 cm, 15 cm, and 20 cm resulted in a coefficient of determination of 0.996, 0.996, and 0.994, respectively. The reduction in rutting depth was about 95%, 91%, and 85%, respectively.

### 4.5. Comparison of IRI and Rutting Depth Measured by MLS and Profilometer

#### 4.5.1. Comparison of IRI

**Figure 17:**
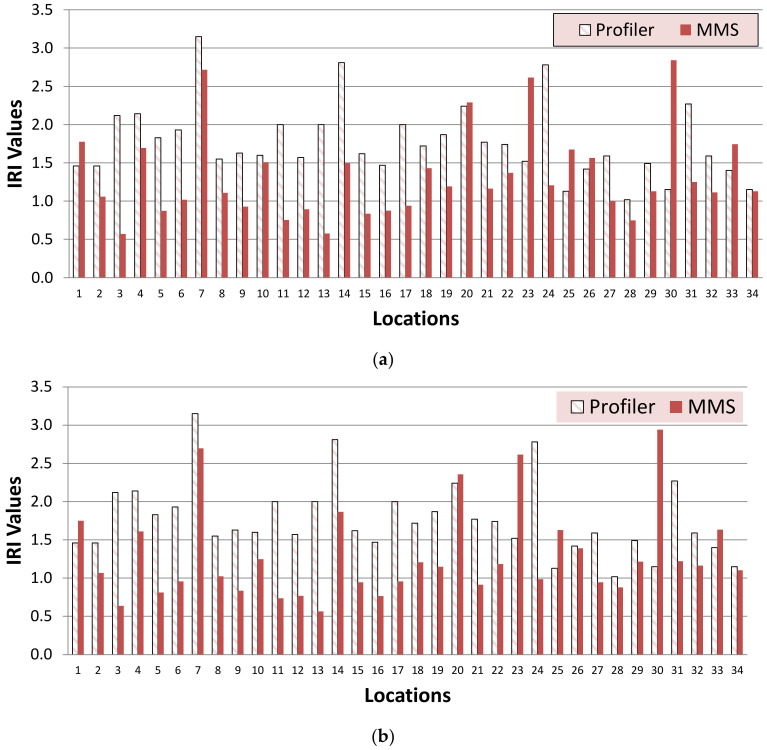
Comparison of IRI values measured by MLS and profilometer for (**a**) 5 cm mesh size and (**b**) 10 cm mesh size.

Figure 17a,b compare the IRI values measured by MLS and profilometers for mesh sizes of 5 cm and 10 cm, respectively. It can be seen that the IRI values are generally higher for both sizes of meshes, i.e., 5 cm and 10 cm compared to the MMS values. However, at some points, the IRI values were observed as less than the MMS values. Further studies are recommended in this direction to explore the proper reasons for these observed results.

#### 4.5.2. Comparison of Rutting Depths

**Figure 18:**
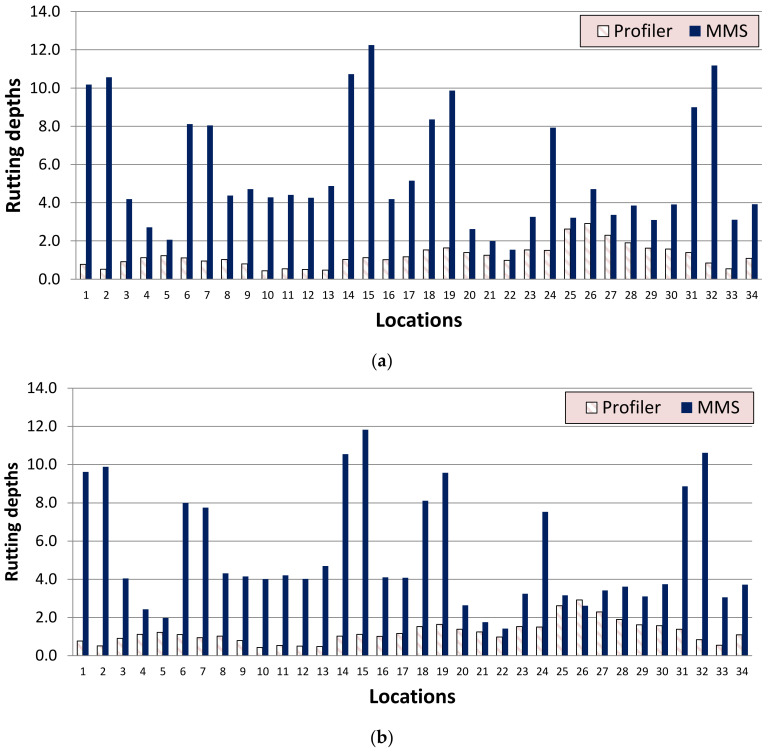
Comparison of rutting depths measured by profilometer and MLS for mesh sizes of (**a**) 5 cm and (**b**) 10 cm.

Like IRI, a comparison was made between the rutting depths measured by the profilometer and MLS. Figure 18a,b present a comparison of rutting depths for mesh sizes of 5 cm and 10 cm, respectively. According to the Department of Highway standard in Thailand, a rutting depth of 6 mm to 12 mm is considered “low”. It is observed that the rutting depth measured by MLS is much higher than the rutting depth measured by a profilometer.

### 4.6. Uncertainty Analysis

Uncertainty analysis aims at quantifying the variability of the output that is due to the variability of the input. The quantification is most often performed by estimating statistical quantities of interest such as mean, median, and population quantiles. In this study, uncertainty analysis is performed for both IRI and rutting depths by using the following equation:

(1) $$UA=\sqrt{\frac{\sum {\left(Xi-u\right)}^{2}}{n*\left(n-1\right)}}$$

where *Xi* = *i*th reading in the data set, *u* = mean of data set and *n* = number of readings. 

The results are shown in Table 2. It can be seen that the uncertainty values are lower for both the IRI and rutting depths as compared to the MMS measurements.

## 5. Conclusions

By technical collaboration with a Japanese expressway company, a mobile mapping 3D laser scanner (MLS) (Trimble MX-8) was introduced to Thailand. MLS is an ideal tool for geometry surveys. However, the performance of MLS needs to be examined. The procedure of measurement, data handling, data accuracy, and data exportation was studied by field measurements on the Thailand expressway. The following important conclusions can be drawn.

1. The MLS measurement can be performed within a 20 km radius of the base station. Thus, a single base station was established in the Bangkok center. MLS measurement took a short time (5 days for 5 locations), but data preparation needed some time. Data must be rectified for correct position, clean unwanted points, and combine several scanning data. This data registration process took 2–3 months. Two kilometers of road scanning produced about 33.5 GB of data. So, enough storage and powerful point cloud management software should be prepared in advance.
2. A good agreement between elevations of the Rama XI bridge in Bangkok measured by point cloud data using MMS and by RTK was obtained. It was proven that MMS is a fast and accurate acquisition method to gather structure geometry that can be used for the health monitoring system.
3. From the point cloud data, bridge profile, twist angles of the deck, and lateral movements of pylons were assessed. The results showed that most of the measured twist angles were within the acceptable limits (±0.05°). No sign of a permanent twist was observed for the deck. The top movement of the west and east pylon was 104 mm and 138 mm, respectively. However, a periodic inspection was suggested due to the absence of initial readings of the pylon.
4. Point cloud needs to be meshed during input file creation. A mesh size of 5 cm × 5 cm is the finest. A mesh size of 10 cm × 10 cm reduced computation time by 75% but reduced the accuracy slightly. Three more mesh sizes, 10 cm × 10 cm, 15 cm × 15 cm, and 20 cm × 20 cm were studied. The results obtained were compared to the mesh size of 5 cm × 5 cm. It was found the mesh size of 10 cm, 15 cm, and 20 cm resulted in $R^{2}$ values of 0.992, 0.979, and 0.969 when compared with the results of the mesh size of 5 cm. A mesh size of 10 cm resulted in a reduction of IRI by 95%.
5. IRI values by profilometer were used as a benchmark. In order to convert the IRI value obtained by MLS to IRI values obtained by a profilometer, empirical correlations between the two methods were proposed. An $R_{2}$ of up to 0.933 for the value of IRI over 1.50–3.0 m/km was obtained from the comparison of IRI values obtained by MLS and profilometer. To convert the IRI value obtained from MLS to the corresponding profilometer value, a conversion value of 0.7597 should be used.
6. It was found that the rutting values obtained from MLS measurements were significantly higher than those obtained from the profilometer.

## Figures and Tables

**Table 1:** The bridge profile data (m).

Length of Bridge	RTK	MLS	Differences
0	0.00	0.00	0.000
36	1.83	1.87	0.037
77	3.94	4.02	0.079
116	5.78	5.89	0.116
193	9.22	9.41	0.184
263	11.06	11.28	0.221
350	12.11	12.35	0.242
397	12.11	12.35	0.242
478	11.22	11.45	0.224
589	8.11	8.27	0.162
672	4.17	4.25	0.083
0	0.00	0.00	0.000

**Table 2:** Uncertainty analysis results.

	IRI Values			Rutting Depth Values		
	Profilometer	MMS 5	MMS 10	Profilometer	MMS 5	MMS 10
UA	0.0822	0.0991	0.1026	0.1000	0.5271	0.5151
